# Facts for the Natives

**Published:** 1863-03

**Authors:** 


### FACTS FOR THE NATIVES.

It is a matter of envious remark, and ought to be one of grave reflection, that foreigners "succeed" better than Americans. In our cities and large towns, both on the seaboard and on the great rivers of the interior, the rule is, that foreigners are going up, and that native citizens are going down, as to the extent of their business, their pecuniary resources, their social position, and influence. In Cincinnati and St. Louis, it is the Dutchman who owns the corner buildings. In New York City, it is the Dutchman who is able to rent or own the corner groceries. Germans and Englishmen here wield the most capital. Our heaviest importers, our very largest commission-houses, have Europeans at their head. We can count in this city alone, foreigners by the dozen, Scotchmen, Irishmen, Germans, English, and others, who, in their youth, were without a dollar, and thousands of miles from home and friends and kindred, and, at the end of thirty years, are to-day worth their hundreds of thousands, and some estimate their fortune at millions.

On the other hand, there are multitudes of the children of native-born citizens who had fortunes to begin with, but who are now bankrupt in money, in character, and in influence; and, thriftless and idle, are going down to an early, or besotted grave.

What makes this wide difference? The poor foreigner comes here with a vivid sense of the evils and the degradation of poverty; he feels that money gives power and influence and position; and, being free to make it, he is willing to work and to save; he is industrious, self-denying and frugal, and the result is, that he rises from the very first hour that his foot touches Castle Garden. It matters not what his position was at home, he embraces the very first opportunity of earning a penny; and, if he cannot get wages at first, he will work for his board, until he can look around, or make his employer feel his worth.

Some years ago, a German youth waited on us at the Pearl Street House, in Cincinnati. There was a neatness in his clothing, an elegance in the arrangement of his hair, and an expression of countenance which indicated elevation; these, with the remarkable promptness with which he attended to his duties, induced us to make inquiries, with the result, of finding that he was willing to be a waiter, rather than spend what little money he had for board, while he was in search of a place. Three years later, he was an active partner in a dry goods establishment, in Fifth street, near Main, when we left the city, and lost sight of him.

For near two years, a young man served us with milk, in Irving Place; he spoke English very imperfectly. We, at length, found that he was a good Hebrew and Greek scholar, and an educated Pharmacien, having spent seven years in learning the art of the apothecary. Having, at length, learned to speak English pretty well, he obtained a profitable berth in his proper calling, and, no doubt, will do well.

Our public places are being rapidly filled with foreigners. Five years ago, there was not a single German conductor on the Fourth Avenue cars, which lead up to the "regal region" about Union Square, Irving Place, and Gramercy Park; to-day, there are scarcely three Americans left—nearly all are Foreign; and, if the reason were inquired into, we are certain it would be found on the score of wages. Americans want to live like princes; to do so, they must be paid like princes; and, in default of a good salary, they throw up their situations, and knock around, dressed in their best, in order to make a good impression, and thus secure a good berth. But, before they know it, their money is exhausted; their clothing begins to look seedy; and with that they begin to feel mean—and who does not, with a bad hat and not a penny in his pocket? and the last step is to turn to politics, for a "place."

These things merit the consideration of reflecting men; and, until a better remedy is found, let the young be instructed, girls as well as boys, that honest labor is a duty—that idleness, helplessness, and thriftlessness, is a disgrace—and that poverty, with pride against work, is a crime against oneself and against society.

